# Supplementary figures and images for: A Myt1 family transcription factor defines neuronal fate by repressing non-neuronal genes
Source: eLife. 2019 Aug 6;8:e46703. doi: 10.7554/eLife.46703 (PMC6684318; doi:10.7554/eLife.46703)

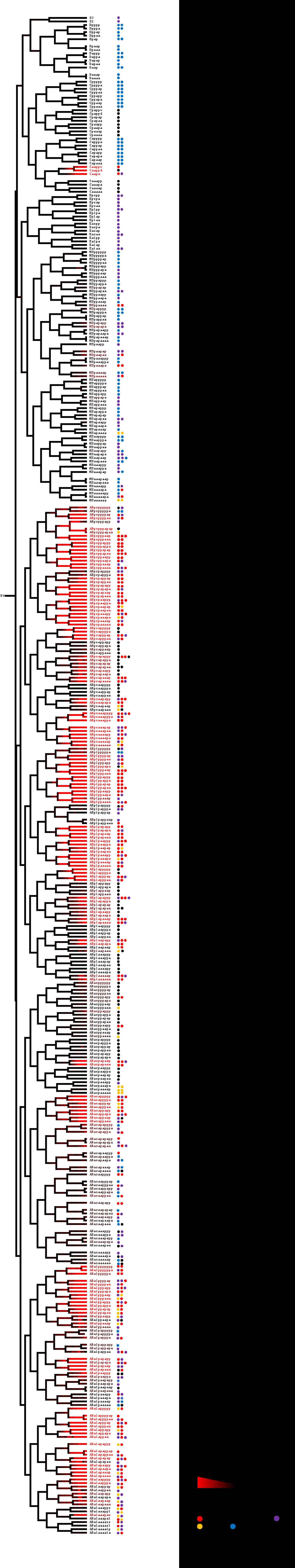

Supplement: Supplementary file 1. [file elife-46703-supp1.jpg]
